# Thermal tolerance of Mediterranean marine macrophytes: Vulnerability to global warming

**DOI:** 10.1002/ece3.4663

**Published:** 2018-11-11

**Authors:** Ioannis Savva, Scott Bennett, Guillem Roca, Gabriel Jordà, Núria Marbà

**Affiliations:** ^1^ Global Change Research Group Institut Mediterrani d’Estudis Avançats (CSIC‐UIB) Esporles Spain; ^2^ Marine and Environmental Research (MER) Lab Limassol Cyprus; ^3^ Marine Ecosystem Dynamics Group Institut Mediterrani d’Estudis Avançats (CSIC‐UIB) Esporles Spain; ^4^ Instituto Español de Oceanografía (IEO) Centre Oceanogràfic de Balears Palma Spain

**Keywords:** activation energy, climate change, experiment, macroalgae, seagrass, thermal limits, thermal sensitivity

## Abstract

The Mediterranean Sea is warming at three times the rate of the global ocean raising concerns about the vulnerability of marine organisms to climate change. Macrophytes play a key role in coastal ecosystems, therefore predicting how warming will affect these key species is critical to understand the effects of climate change on Mediterranean coastal ecosystems. We measured the physiological performance of six dominant native Mediterranean macrophytes under ten temperature treatments ranging from 12 to 34°C to examine their thermal niche, and vulnerability to projected warming in the western Mediterranean up until 2100. Among the macrophytes tested, *Cymodocea nodosa* was the species with the highest thermal optima and it was beyond current summer temperature. Therefore, *C. nodosa* may benefit from projected warming over the coming century. The optimal temperature for growth of the other species (*Posidonia oceanica*,* Cystoseira compressa, Padina pavonica*,* Caulerpa prolifera,* and *Halimeda tuna*) was lower. Similarly, the species presented different upper lethal limits, spanning at least across 5.1°C between 28.9°C (*P. oceanica*) and >34°C (*C. nodosa*). Our results demonstrate the variable physiological responses of species within the same local community to temperature changes and highlight important potential differences in climate change vulnerability, among species within coastal marine ecosystems.

## 1 INTRODUCTION

Global warming is a major driver of marine biodiversity change (Poloczanska et al., 2013). Organisms are responding to climate warming by either tolerating change (i.e., through phenotypic plasticity; Somero, 2010), adapting (i.e., through genetic variation within the population), or migrating to more favorable environments (Pecl et al., 2017; Poloczanska et al., 2013). Species redistribution has been observed across the biosphere (Pecl et al., 2017; Poloczanska et al., 2013) and will be crucial to the survival of individual species at a global scale. Locally, the disappearance of native species from a community can have catastrophic impacts to the resilience and function of that ecosystem (Bennett, Wernberg, Joy, Bettignies, & Campbell, 2015; Wernberg, Bennett, et al., 2016a). Moreover, high rates of warming, increasing frequency and magnitude of extreme temperature events, and the long generation time of many species may exceed the capacity of many species to adapt to current rates of change.

Marine macrophytes (seaweeds and seagrasses) are foundation species, vital to the structure and function of benthic marine ecosystems, and are among the most productive coastal communities in the world (Christie, Norderhaug, & Fredriksen, 2009; Mann, 1973). Furthermore, macrophytes provide important ecosystem services (Bennett et al., 2016; Costanza et al., 2014) including coastal protection, carbon sequestration, nutrient cycling, and food production upon which human society closely depends (Bennett et al., 2016; Christie et al., 2009; Dhir, 2015; Mcleod et al., 2011; Mineur et al., 2015). There is increasing evidence demonstrating the in situ responses of macrophytes to warming across the globe (e.g., Lima, Ribeiro, Queiroz, Hawkins, & Santos, 2007; Wernberg et al., 2011; Marbà & Duarte, 2010; Nicastro et al., 2013; Wernberg, Bettignies, Joy, & Finnegan, 2016b); nevertheless, the fundamental thermal niche remains untested for most species (but see Martínez, Arenas, Trilla, Viejo, & Carreño, 2014; Wiencke, Bartsch, Bischoff, Peters, & Breeman, 1994). Understanding how marine macrophytes respond to temperature across the breadth of their current thermal niche and future thermal conditions is crucial in order to anticipate the possible impacts of climate change (Dell, Pawar, & Savage, 2011), particularly in areas that exhibit rapid warming rates. A well‐documented case is the Mediterranean Sea, which is warming threefold faster than the global ocean (Burrows et al., 2011; Vargas‐Yáñez et al., 2008) while the occurrence and the duration of extreme temperature events have increased by 200%–500% over the past 60 years (Diffenbaugh, Pal, Giorgi, & Gao, 2007; Vargas‐Yáñez et al., 2008, 2010).

The thermal response of any biological activity follows a performance curve within the thermal niche (Nati, Lindstrom, Halsey, & Killen, 2016). An organism's thermal sensitivity is characterized by the rate of change in physiological performance in response to a degree of temperature change. Beyond a species optimal temperature, high thermal sensitivity will result in a rapid decline in fitness with increasing temperatures, until an upper lethal temperature limit is reached. The difference in temperature between an organism's upper thermal limit and the upper environmental temperature is defined as the “Thermal Buffer” (Bennett et al., 2015). The vulnerability of an organism to warming is the time required for warming temperatures to erode the thermal buffer of an organism to reach its upper lethal thermal limit.

Within any given biological community, ambient warming rates (i.e., exposure) will be the same among organisms; however, sensitivity and vulnerability to warming may vary among species due to intrinsic differences in the thermal niche. The variation in thermal sensitivity and thermal buffer among co‐occurring species remains poorly resolved due to a lack of understanding about the drivers of thermal limits at the population level. The thermal limits of a population may occur along a spectrum from highly “locally adapted” (i.e., reflecting the climate conditions within the local environment) to “highly conserved” (i.e., reflecting the thermal limits and global distribution of the species). Thermal limits of locally adapted populations will be shaped by local climatic conditions and will therefore vary under different climatic conditions throughout a species geographical range. By contrast, thermal limits will not differ among conspecific populations that exhibit a conserved thermal niche and instead may be best characterized by the thermal extremes observed in the warmest and coolest locations of the species global distribution. Phylogeographic legacies, disturbance history, population connectivity, and life‐history traits may all contribute to the type of thermal niche populations exhibit. In the context of climate change, these processes could result in similarities or differences in species sensitivity to warming within any given ecosystem and provide insights into where adaptive management strategies could take place to boost the resistance of populations that are threatened by warming (Bennett et al., 2015).

Here, we assess the thermal sensitivity and vulnerability of six marine macrophyte species from a coastal Mediterranean community. Specifically, we examine the thermal physiological response of two seagrass species (*Posidonia oceanica and Cymodocea nodosa*) and four seaweed species (*Cystoseira compressa*,* Caulerpa prolifera*,* Halimeda tuna,* and *Padina pavonica*) across the breadth of their current and future thermal range. To this aim, we further examined how thermal buffers vary among species living within the same community and whether thermal limits of the species reflect the local climatic regime (potentially reflecting locally adapted/acclimatized populations known as the fundamental thermal niche) or reflect the global climatic distribution of the species (indicating a more conserved thermal niche).

## 2 MATERIALS AND METHODS

### 2.1 Study location, species selection, and sampling procedure

**Figure 1:**
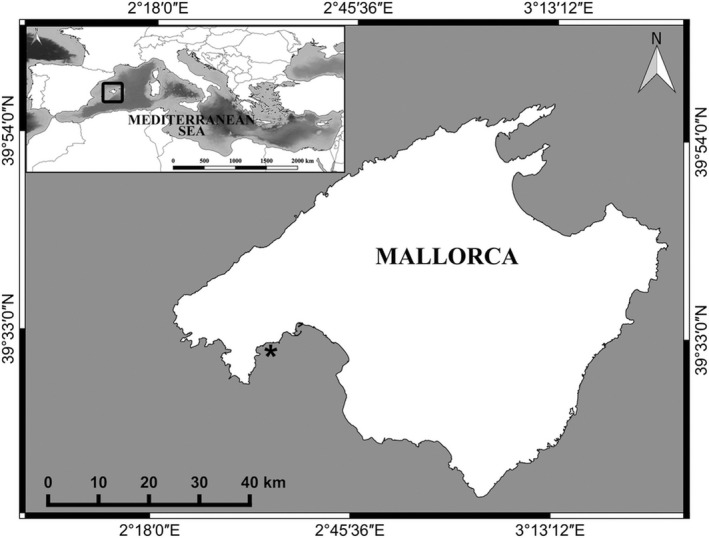
Location of experimental macrophyte donor populations in south‐western Mallorca Island (Mediterranean Sea)

The study was conducted in Mallorca, Balearic Islands, western Mediterranean Sea (Figure 1; Supporting Information Figure S1), where summer sea surface temperatures (SST) have warmed by 1.15°C over the past three decades (Marbà, Jordà, Agustí, Girard, & Duarte, 2015). Currently, sea surface temperatures in Balearic region ranges between 13°C and 27.5°C with minimum temperature recorded during February‐March and maximum temperature during August (Marbà & Duarte, 2010; Samperio‐Ramos, Olsen, Tomas, & Marbà, 2015). However, the area experiences heat waves and strong water stratification during the summer (Coma et al., 2009; Marbà & Duarte, 2010), which can lead to high temperatures that exceed 28°C down to 30–40 m depth (López‐Jurado, Marcos, & Monserrat, 2008). An ensemble of 12 regional and global atmospheric‐ocean circulation models projects that western Mediterranean SST warming will continue during the 21st century, SST increasing by 2°C by 2050 and by 3.4°C by the end of this century under an scenario of moderate greenhouse gas emissions (SRES A1B or equivalently RCP 6.0; Jordà, Marbà, & Duarte, 2012).

Thermal tolerance experiments were conducted using six Mediterranean macrophyte species, consisting of two seagrasses (*P. oceanica* (Linnaeus) and *C. nodosa* (Ucria) Ascherson) and four seaweeds (*C. compressa* (Esper) Gerloff & Nizamuddin, *C. prolifera* (Forsskål) J.V. Lamouroux*, P. pavonica* (Linnaeus) Thivy, and *H. tuna* (J.Ellis & Solander) J.V. Lamouroux). The chosen species were selected because of their ecological importance, they are canopy‐forming species, and they have wide distribution within the Mediterranean. *P. oceanica* is endemic to the Mediterranean Sea. *C. nodosa* is present in the Mediterranean Sea and extends throughout the coast of West Africa, north to mid Portugal (including Madeira Island), Canary and Cape Verde Islands (Short et al., 2010). *Cystoseira compressa* is a common brown seaweed inhabiting rocky shores of the Mediterranean coasts at 0–1 m depth and plays a central role as an engineering species on rocky shores (Gianni et al., 2013; Thibaut, Pinedo, Torras, & Ballesteros, 2005). *Caulerpa prolifera* is distributed across subtropical and tropical regions (Varela‐Álvarez et al., 2015). *Padina pavonica* and *H. tuna* are calcifying brown and green seaweeds, respectively, and are widely distributed throughout temperate and tropical waters of Atlantic and Indo‐Pacific, as well as encompassing populations in the Mediterranean Sea in littoral habitats (Hillis‐Colinvaux, 1980; Silberfeld et al., 2013). Both genera are major contributors in sediment formation through calcium carbonate (CaCO_3_) deposition in shallow waters of tropical and subtropical regions (Wefer, 1980).

All specimens were sampled within three enclosed bays in southwest Mallorca, Balearic Islands (Figure 1; Supporting Information Figure S1) at a depth range of 1–5 m during February for *C. nodosa* and *P. oceanica*; March for *C. compressa* and *C. prolifera*; April for *H. tuna* and *P. pavonica* in 2016. The in situ SST during sampling was 18°C in February and 17°C in March and April, thus during the coldest seawater season. Hence, we performed the experiments when the specimens were acclimated to winter conditions, particularly *P. oceanica* and *C. nodosa*, when they may have lower upper thermal limits than during summer. Our experiments coincided with the period of increasing seasonal production, and thus, experimental macrophytes were in healthy condition.

The plant material was submerged in seawater within cool boxes and was transported to the laboratory, where all specimens were left to acclimatize at laboratory conditions for two days at their in situ temperature with air supply and a 12 hr light: 12 hr dark photoperiod, inside a temperature‐controlled chamber.

### 2.2 Experimental design

Following the initial acclimatization period, each specimen was transferred to individual experimental aquaria (Supporting Information Figure S2), which consisted of a double‐layered transparent plastic bag filled with 1 L of filtered seawater (60 μm), except for *P. oceanica* where 2 L of filtered seawater was used due to longer leaves. Experimental bags were suspended within 150‐L temperature‐controlled baths filled with freshwater. In total, ten baths were used, one for each experimental temperature treatment. Bath temperatures were initially set to the acclimatization temperature (i.e., in situ temperatures) and were subsequently increased or decreased by 3°C every 24 hr until the desired experimental temperature was achieved. Experimental temperatures were 12, 15, 18, 21, 24, 26, 28, 30, 32, and 34°C. For each species, seven replicated experimental bags with one seagrass shoot/algal fragment per bag were deployed in each temperature treatment. Seagrass replicates contained a single shoot (including leaves and vertical rhizome) with roots and, for *C. nodosa*, the shoot was attached to two internodes of horizontal rhizome. The algal replicates encompassed the holdfast, in the case of *P. pavonica*,* C. compressa,* and *H. tuna*, or 2–3 fronds attached to a stolon with rhizoids in the case of *C. prolifera*. All specimens had a young growing tissue. Once the targeted temperatures were reached in all of the baths, experiments ran for 14 days for the four, faster‐growing, algal species and 21 days for the, slower‐growing, seagrasses to allow for measurable growth in all species at the end of the experiment. The experimental setup was run three times in total, thus, each bath contained incubated plastic bags of two species at a time, within a temperature‐controlled chamber at an ambient air temperature of 10°C (Supporting Information Table S1). Bags were randomly distributed within the baths, suspended with their surface kept wide open to allow gas exchange, and were illuminated with a 12 hr light:12 hr dark photoperiod through fluorescent aquarium growth lamps. The water within the bags was kept mixed by stirring the baths with aquaria pumps. The light intensity throughout each bag was measured via a photometric bulb sensor (LI‐COR) and ranged between 180 and 258 μmol m^−2^ s^−1^. The temperature in the baths was controlled and recorded with an IKS‐AQUASTAR system, which was connected to heaters and thermometers. The seawater within the bags was renewed every 4 days, and salinity was monitored daily through an YSI multiparameter meter. Distilled water was added when necessary to ensure salinity levels remained within the range of 35–38 PSU. At the end of the experiment, we assessed the species growth and survival responses to the experimental thermal range. We also examined the performance curves of maximum quantum yield (MQY) across the experimental thermal range of the experimental macrophytes at the end of the experiment. Because thermal responses of MQY were similar to those of growth, MQY results are provided in Supplementary Material (Supporting Information Table S2; Supporting Information Figures S3 and S4).

### 2.3 Growth rate

The growth was calculated as the relative growth rate (RGR, day^‐1^) as described by Hunt (1990):

$$\text{RGR}\;\left({\text{day}}^{-1}\right)=\frac{\ln\;\text{DW}2-\ln\text{DW}1}{t}$$

where, DW1 and DW2 are the dry weights at the beginning and the end of the experiment (Supporting Information Table S3), respectively, and *t* is the experiment duration in days. RGR allows for comparison of growth rates of photosynthetic organisms across a wide range of sizes (Nielsen, Enriquez, Duarte, & Sand‐Jensen, 1996).

The surface of the experimental fragments was blotted and air‐dried with care avoiding damaging them. The wet weight (g WW) of these fragments was then recorded at the beginning and end of each experiment (*n* = 7 for each temperature treatment). At the end of the experiment, the fragments from each treatment were oven‐dried at 80°C for 24 hr to obtain dry weights. The initial and final dry weights were estimated from a conversion factor through a linear relationship between wet and dry weights of each species (*n* = 70).

### 2.4 Survival

Survival was assessed at the end of the experiment as the percentage of treatment replicated specimens presenting photosynthetic tissue necrosis for seaweed species (e.g., transparency in *C. prolifera* and whitening in *H. tuna*) and *C. nodosa* or meristem mortality for *P. oceanica*.

### 2.5 Performance curve, thermal sensitivity, and vulnerability

The responses of biological traits to temperature are characterized by a bell‐shaped relationship across the thermal breadth whereby an organism operates and the biological activity of a species below and beyond its optimal temperature reflects the thermal sensitivity (Dell et al., 2011). Therefore, the performance curves of the RGR (and MQY in Supplementary Material) of all six macrophytes were described by fitting the temperature cardinal model with inflexion (CTMI) described by (Ras, Steyer, & Bernard, 2013):

$$\begin{array}{c} P_{\max}=P_{\text{opt}}\frac{\left(T-T_{\max}\right){\left(T-T_{\min}\right)}^{2}}{\left(T_{\text{opt}}-T_{\min}\right)\left[\left(T_{\text{opt}}-T_{\min}\right)\left(T-T_{\text{opt}}\right)-\left(T_{\text{opt}}-T_{\max}\right)\left(T_{\text{opt}}+T_{\min}-2T\right)\right]}\\ \text{for}\;T_{\min}\leq T\leq T_{\max} \end{array}$$

where, *T* is the temperature (°C), *P* is the performance of the ecophysiological trait, *T*_opt_ is the optimal temperature at which the ecophysiological trait is maximal, *P*_opt_ is the ecophysiological trait's performance (RGR_opt_, MQY_opt_) at *T*_opt_, *T*_min_, and *T*_max_ are the hypothetical lower and upper critical temperatures (critical thermal limits), respectively, through which the ecophysiological trait's performance is zero. The range between *T*_min_ and *T*_max_ is the tolerance range. The thermal performance breadth (TPB) was also determined and was defined as the temperature range through which the ecophysiological trait performed close to optimally (defined as the 80th percentile of the CTMI fits; Nati et al., 2016).

The thermokinetics of the biological activities are described and quantified by the Van't Hoff‐Arrhenius relation when incorporated into the metabolic theory of ecology (MTE). The thermal sensitivity of each species was assessed by the rate of change of RGR (and MQY in Supplementary Material) per degree C and the activation energy (*Ea*) within the thermal ranges below and beyond the *T*_opt_ of each trait (*T*_opt_ being included in both rising and falling phase of the performance curve). For the purpose of this analysis, the *T*_opt_ used here was derived from the CTMI and rounded to the nearest integer and close to temperatures used in this experiment. The rate of change was estimated as the slope of a linear regression model between the ecophysiological trait and treatment temperature. The *Ea* was estimated using the MTE equation (Brown, Gillooly, Allen, Savage, & West, 2004; Dell et al., 2011):

$$R=R_{o}e^{-Ea/kT}$$

where, *R_o_* is the state‐depended scaling coefficient of the organism, *Ea* is the activation energy, *k* is Boltzmann's constant (8.62 × 10^‐5^ eV/K), *T* is the temperature (in kelvin), and *R* is the biological trait (i.e., RGR, MQY).

Upper lethal limits (LT_50_) of the species were estimated using a logistic growth equation, whereby lethal temperatures accounted for 50% survival of the fitted values of the logistic model:

$$y=\frac{a}{1+{\left(\frac{X}{c}\right)}^{b}}$$

where *a*,* b*, and *c* are the coefficients of the model, *y* is survival (%), and *X* is the experimental temperatures.

### 2.6 Thermal buffer

We compiled distributional data for all six studied macrophytes across the globe from published literature and AlgaeBase ( https://www.algaebase.org/, accessed November 2016). For each of these geographical points, SST (daily at 0.1° spatial resolutions, ~10 km) for the last 35 years (1981–2016) was acquired to obtain the minimum and maximum temperatures at which natural populations of these species are exposed at. In particular, minimum and maximum temperatures were extracted as the 1st and 99th percentiles of SST, respectively. In order to evaluate the difference between the local and global thermal ranges of the studied species’ populations, 1st and 99th percentiles of SST were also extracted for the sampling sites in Mallorca. The assessment of the thermal buffer on the upper thermal limit was carried out comparing the empirically observed LT_50_ and maximum local and global temperatures for each species.

### 2.7 Statistical analysis

The relationship of RGR with temperature was statistically tested on the rise and falling phase of the performance curves through linear regression as part of the warming sensitivity analysis. Assumptions for normality and equal variances were verified using the Anderson‐Darling and Bartlett tests, respectively. When assumptions of normality or homogeneity were not met, data were transformed (log_10_ or square root). The level of significance *α* was adjusted to 0.05 for all statistical analyses, and all statistical analyses were conducted in R (R core team). All graphics were generated with R‐studio, package: ggplot2 (Wickham, 2009).

## 3 RESULTS

### 3.1 Performance curves and sensitivity

**Figure 2:**
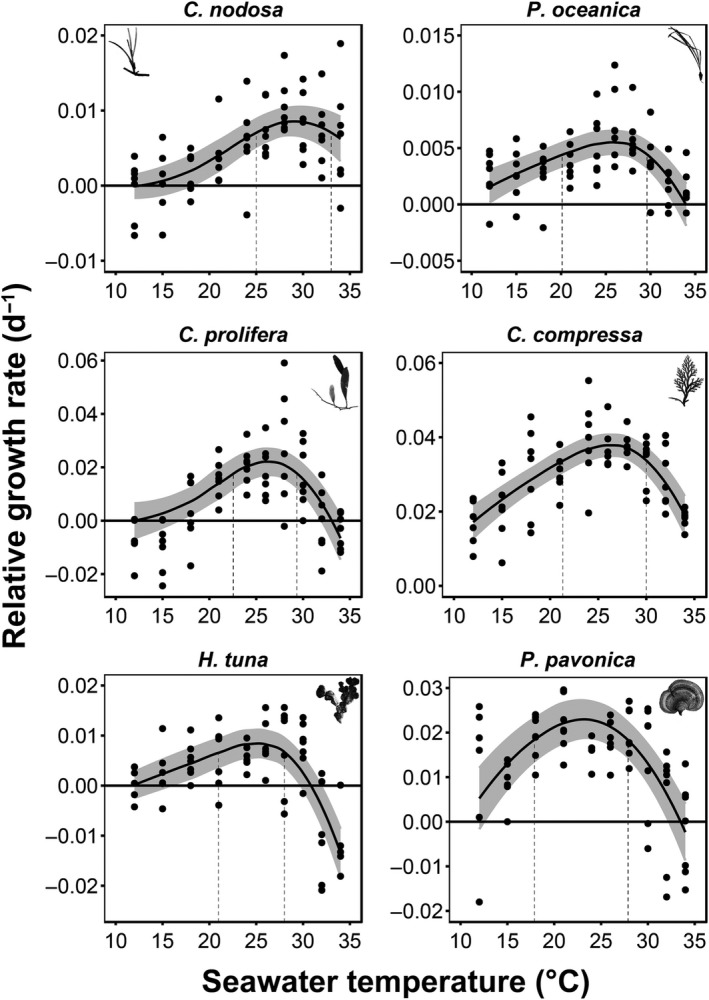
The bell‐shaped RGR responses of all six macrophyte species to experimental seawater warming fitted with the CTMI. The shaded area indicates the upper and lower 95% confidence intervals. Vertical dotted lines illustrate the TPB

Temperature had a significant effect on the RGR for all six macrophytes both at the rising and falling phases of the thermal response curves (Table 1; Figure 2), with only one exception at the falling phase of *C. nodosa*, where temperature had no effect on the RGR (Table 1; Figure 2).

**Table 1:** The temperature‐associated parameters (*T*_opt_, *T*_min_, *T*_max_, RGR_opt_) ±*SE* obtained by the temperature cardinal model with Inflexion (CTMI), thermal performance breadth (TPB), the rate of change per degree of Celsius ±*SE* (rate of rise, rate of fall), and activation energy (*Ea*_rise_, *Ea*_fall_) ± *SE* of the rising and falling phase of the bell‐shaped RGR responses for all six species

Temperature parameters	*Cymodocea nodosa*	*Posidonia oceanica*	*Caulerpa prolifera*	*Cystoseira compressa*	*Halimeda tuna*	*Padina pavonica*
*T*_opt_ (°C)	29.5 ± 1.3**	25.8 ± 1.3**	26.4 ± 0.9**	26.4 ± 0.9**	25.1 ± 1.0**	23.1 ± 1.3**
*T*_min_ (°C)	12.9 ± 4.1**	*N*/A	13.5 ± 2.1**	*N*/A	9.7 ± 4.6*	*N*/A
*T*_max_ (°C)	36.9 ± 2.9 **	33.8 ± 0.5**	33.09 ± 0.4**	36.1 ± 0.9**	31.1 ± 0.5**	33.5 ± 0.6**
RGR_opt_	0.01 ± 0.001**	0.005 ± 0.0005**	0.02 ± 0.002**	0.04 ± 0.001**	0.01 ± 0.001**	0.02 ± 0.002**
TPB (°C)	25–33	20.1–29.6	22.6–29.3	21.3–30	21.9–28	17.9–27.9
Rate of rise (°C^−^^1^)	0.0006 ± 0.00009 (*R* ^2^ = 0.46; *p < *0.05; *N* = 56)	0.0003 ± 0.00008 (*R* ^2^ = 0.52; *p < *0.05; *N* = 43)	0.0023 ± 0.00030 (*R* ^2^ = 0.60; *p* < 0.05; *N* = 43)	0.0015 ± 0.00030 (*R* ^2^ = 0.43; *p < *0.05; *N* = 43)	0.0005 ± 0.00020 (*R* ^2^ = 0.21; *p* < 0.05; *N* = 36)	0.0015 ± 0.00050 (*R* ^2^ = 0.24; *p* < 0.05; *N* = 35)
Rate of fall (°C^−^^1^)	−0.0004 ± 0.00070 (ns)	−0.0009 ± 0.00008 (*R* ^2^ = 0.47; *p* < 0.05; *N* = 36)	−0.0036 ± 0.00080 (*R* ^2^ = 0.36; *p < *0.05; *N* = 36)	−0.0023 ± 0.00040 (*R* ^2^ = 0.53; *p < *0.05; *N* = 36)	−0.0020 ± 0.00040 (*R* ^2^ = 0.40; *p < *0.05; *N* = 43)	−0.0025 ± 0.00040 (*R* ^2^ = 0.44; *p < *0.05; *N* = 43)
*Ea*_rise_ (eV)	0.968 ± 0.150 (*R* ^2^ = 0.42; *p* < 0.05; *N* = 50)	0.368 ± 0.114 (*R* ^2^ = 0.45; *p* < 0.05; *N* = 40)	2.209 ± 0.218 (*R* ^2^ = 0.68; *p* < 0.05; *N* = 31)	0.442 ± 0.085 (*R* ^2^ = 0.40; *p* < 0.05; *N* = 43)	0.922 ± 0.289 (*R* ^2^ = 0.23; *p* < 0.05; *N* = 31)	0.427 ± 0.178 (*R* ^2^ = 0.14; *p* < 0.05; *N* = 33)
Ea_fall_ (eV)	0.455 ± 0.848 (ns)	1.456 ± 0.236 (*R* ^2^ = 0.53; *p < *0.05; *N* = 30)	2.049 ± 0.437 (*R* ^2^ = 0.38; *p < *0.05; *N* = 27)	0.667 ± 0.103 (*R* ^2^ = 0.56; *p < *0.05; *N* = 36)	1.794 ± 0.423 (*R* ^2^ = 0.28; *p < *0.05; *N* = 31)	1.113 ± 0.275 (*R* ^2^ = 0.28; *p < *0.05; *N* = 36)

Note

Asterisks indicate the significance of the CTMI parameters’ estimates (**p < 0.01, *p < 0.05).

ns: not significant; N/A: not applicable, indicating unrealistic estimated values due to the lack of further empirical data below 12°C.

The thermal optima (*T*_opt_) in *C. nodosa* was recorded at 29.5°C (the highest observed in this study) and the thermal performance breadth (TPB) of RGR was located on the upper range of the temperatures tested, between 25 and 33°C (Table 1; Figure 2). Species that also performed particularly well in warmer temperatures were *C. compressa* and *C. prolifera*, both exhibiting the *T*_opt_ at 26.4°C and the TPB ranging between 21.3 and 30°C and 22.6 and 29.3°C, respectively (Table 1; Figure 2). Slightly wider TPB's were observed in *P. pavonica* (17.9–27.4°C) and *P. oceanica* (20.1–29.6°C), whereas *H. tuna* displayed the narrowest one (21.9–28°C; Table 1; Figure 2). The RGR *T*_opt_ for *P. pavonica* peaked at 23.1°C (the lowest observed in this study), while for *P. oceanica* and *H. tuna* at 25.8°C and 25.1°C, respectively (Table 1).

The RGR of *C. compressa* increased 2.7‐fold and ~1.3‐fold faster per degree of warming than *H. tuna* and the rest of the macrophytes, respectively, before reaching their *T*_opt_ (Table 1). *C. prolifera* and *P. pavonica* ranked at the second and third place, respectively, followed by the seagrasses and lastly by *H. tuna*, which exhibited the slowest rate of increase in the RGR. On the other hand, sharp RGR decline beyond the *T*_opt_ was evident in *P. oceanica*, being 3.61‐fold and ~1.45‐fold faster per degree of warming than *C. nodosa* and the rest of the macrophytes, respectively (Table 1).

**Figure 3:**
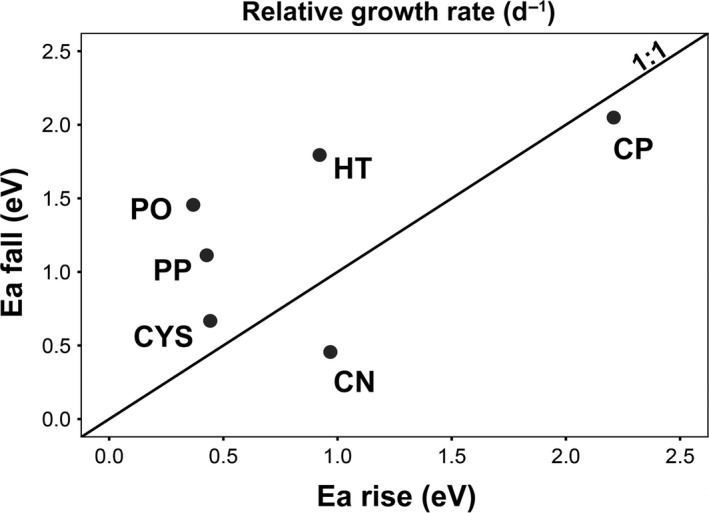
The activation energy at the falling phase (*Ea*_fall_) against the rising phase (*Ea*_rise_) of all six macrophytes for RGR. The black diagonal line represents a 1:1 ratio line. PO: *Posidonia oceanica*, CN: *Cymodocea nodosa*, CYS: *Cystoseira compressa*, CP: *Caulerpa prolifera*, HT: *Halimeda tuna* and PP: *Padina pavonica*

*Caulerpa prolifera* was the most temperature‐dependent species, attaining the highest activation energy of RGR in both rising and falling phases (Table 1; Figure 3). *Cymodocea nodosa* was another species for which growth was driven by temperature changes below *T*_opt_. High sensitivity to warming below and beyond *T*_opt_ was noticeable in *H. tuna*. *C. compressa, P. oceanica*, and *P. pavonica* displayed relatively low sensitivity to temperature changes in RGR below *T*_opt_, but the latter two species revealed high sensitivity to warming in the falling phase (Table 1; Figure 3).

### 3.2 Thermal vulnerability and thermal buffer

**Figure 4:**
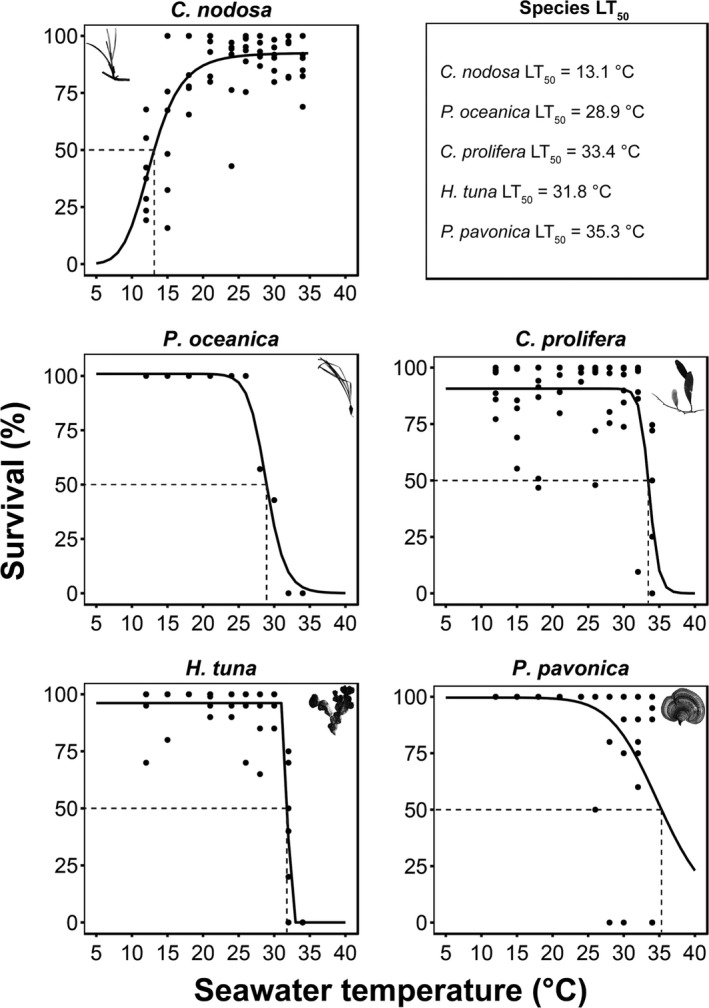
The survival curves for five macrophyte species across an extended temperature range from the one used in the experiment for the purpose of the model fitting. The lethal temperatures (LT_50_, causing a 50% survival decline) acquired from the logistic growth model are indicated

All species but *C. nodosa* were tolerant to the lower temperatures tested, as maximum survival persisted down to 12°C. Conversely, *C. nodosa* survival started to decrease below 18°C and exhibited LT_min50_ at 13.1°C (Figure 4).

The highest tolerance to warming was observed in *C. nodosa*, which was reflected on the health status of the leaves on its upper temperature range tested (Figure 4; Supporting Information Figure S5). Tissue degradation in the remaining macrophytes and meristem mortality in *P. oceanica* took place beyond their RGR *T*_opt_ (Figure 4; Supporting Information Figure S5). For *P. oceanica,* the LT_50_ was observed at 28.9°C (Figure 4). Unexpectedly, the LT_50_ was found lower than RGR's upper TPB limit and *T*_max_, which could reflect high initial growth rates at the beginning of the experiment, despite plant mortality later on. In *P. pavonica*, tissue degradation initiated beyond its optimal temperature, when individuals started sporulation, with 35.3°C identified as its LT_50_ (Figure 4). Sporulation in *P. pavonica* intensified between 26°C and 32°C. *C. prolifera* and *H. tuna* experienced abrupt tissue degradation beyond 30°C and LT_50_ at 33.4°C and 31.8°C, respectively (Figure 4), both exhibiting loss of pigmentation (Supporting Information Figure S5). No mortality or tissue degradation was observed in *C. compressa* other than pigmentation changes toward a darker shade of brown beyond 28°C and became more conspicuous throughout the whole specimens’ body at 30°C, 32°C, and 34°C.

**Figure 5:**
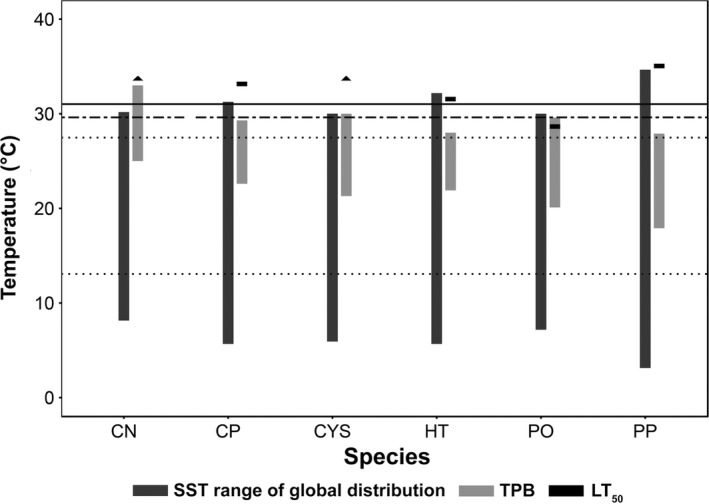
The current range of SST across global species distribution, the TPB (Thermal performance breadth), and the LT_50_ of each species acquired during the experiments. For species where LT_50_ was not identified, an arrowhead is indicated instead, meaning that LT_50_ exceeds this study's maximum experimental treatment (34°C). Current minimum and maximum SST (dotted lines; 13–27.6°C), projected SST by 2050 (dashed line; 29.6°C), and projected SST by 2100 (solid line; 31°C) in Mallorca are shown. PO: *Posidonia oceanica*, CN: *Cymodocea nodosa*, CYS: *Cystoseira compressa*, CP: *Caulerpa prolifera*, HT: *Halimeda tuna* and PP: *Padina pavonica*. Projected SST values from Jordà et al., (2012)

The upper lethal temperatures (LT_50_) of four species (*C. nodosa*,* C. prolifera*,* C. compressa* and *P. pavonica*; Figure 5) exceeded upper SST's observed across each species’ global distribution (Supporting Information Table S4). In contrast, upper LT_50_ for *H. tuna and P. oceanica* (Figure 5) were lower than or very close to the maximum SST's recorded within the species’ global distribution, but they still exceeded current local summer SST (Figure 5). Based on upper LT_50_, *P. oceanica* displayed the highest vulnerability, with a thermal buffer of 1.3°C under current conditions, with local SST projected to exceed it by 2050 under a moderate scenario of greenhouse gas emissions (Jordà et al., 2012). Among the other species for which upper LT_50_ could be measured, thermal buffers ranged from 4.2°C for *H. tuna,* 5.8°C for *C. prolifera,* and 7.7°C for *P. pavonica*. Local SST's are not projected to exceed the LT_50_ for these three species before 2100 (Figure 5).

## 4 DISCUSSION

Our results demonstrate variable thermal niche profiles among the Mediterranean macrophytes *C. nodosa*,* P. oceanica*,* C. prolifera*,* C. compressa*,* P. pavonica,* and *H. tuna*. Among the six species tested, *C. nodosa* may positively benefit from warming over coming decades, whereas the remaining five species could potentially be negatively affected by warming. Of those, *P. oceanica* appears the most vulnerable. Our results highlight the variable nature of physiological responses to climate change reflected on species within the same local community, illustrating that further understanding on biotic sensitivity to warming, in conjunction with projections of warming exposure, will be crucial to understand climate change impacts on marine coastal systems. Uncovering the underlying environmental and evolutionary drivers of thermal niche characteristics at the population level will therefore be essential to develop a more generalized understanding of climate change vulnerability.

Despite the populations of the studied macrophytes were exposed to a similar climatic regime in nature, the thermal performance across current and projected temperature range differed substantially among species. This was reflected in optimal temperatures and upper thermal lethal limits spanning across at least 5.1°C and 6.4°C, respectively, and a difference in the amplitude of TPB up to 4°C. This demonstrates that intrinsic differences in the thermal niche contribute to macrophyte sensitivity and vulnerability to warming. Indeed, *T*_opt_ observed in our study were consistent with previous findings for *C. nodosa* and *P. oceanica* from the western Mediterranean (Olsen, Sánchez‐Camacho, Marbà, & Duarte, 2012). Observations on *T*_opt_ for the remaining species have been reported from other regions and did not vary much compared to our study's estimates. For instance, the growth of *C. prolifera* was found to peak at 30°C in the western Mediterranean (Terrados & Ros, 1992), 21°C for *H. tuna* from a 18 m deep population in the north western Mediterranean (Ballesteros, 1991), 24°C for *C. compressa* in northern Adriatic Sea (Falace, Zanelli, & Bressan, 2005), and 21°C for *P. pavonica* in the Red Sea (Mergner & Svoboda, 1977). Variability in species sensitivity to temperature was also evident on the activation energies (both in the rise and fall phases), which fall within those reported for organisms in general (Dell *et al.,* 2011).

Growth and physiology of Mediterranean macrophytes vary seasonally, largely coupled to annual variability of temperature and/or light availability (e.g., Marbà, Cebrián, Enríquez, & Duarte, 1996, Enríquez, Marbà, Cebrián, & Duarte, 2004, Terrados & Ros, 1992, Ballesteros, 1991). Therefore, acclimation of macrophytes is an important condition in experiments such as ours, and the experimental warming rate and magnitude of thermal conditions can have an important effect on lethal limits (Peck, Morley, Richard, & Clark, 2014). Indeed, winter‐acclimated plants may lower the expected *T*_max_, whereas relatively fast acclimation rates may raise *T*_max_, compared to slowly acclimated specimens (Peck et al., 2014). Therefore, the results of our experiment do need to be treated with some caution when compared with other experiments and future thermal conditions. Nevertheless, our findings provide sound comparison between the species examined and our results were consistent with lethal limits observed in natural populations in the field for which data are available (i.e., *Posidonia oceanica*; Marbà & Duarte, 2010; Marbà et al., 2015), providing confidence of their robustness.

Based on a moderate greenhouse gas emissions scenario (SRES A1B or equivalently RCP 6.0), SST in the western Mediterranean Sea is projected to increase, on average, by 2°C by 2050 and by 3.4°C by 2100 resulting in summer average temperature of approximately 29–30.5°C by the end of the century (Jordà et al., 2012). Such seawater temperatures in Mallorca will stretch down to deeper waters, since the summer thermocline is located between to 30 and 40 m depth (López‐Jurado et al., 2008). High *T*_opt_ for *C. nodosa* (29.5 ± 1.3°C) and high thermal sensitivity in the rise phase (*E*a_rise_ = 0.968 ± 0.15 eV) suggest that warming may substantially benefit *C. nodosa* populations over the coming decades. In contrast, summer temperatures currently exceed the observed *T*_opt_ for *C. prolifera, H. tuna,* and *P. oceanica* during the experiment, all of which displayed very high thermal sensitivities in the fall phase, (i.e., *Ea*_fall_ = 1.4–2.1 eV), suggesting that ongoing warming may negatively impact these species. Among these species, our results suggest that *P. oceanica* may be the most vulnerable to warming, given its upper LT_50_ is set at 28.9°C. These findings are also consistent with previous studies that reported that temperatures exceeding 28°C during consecutive heat waves in 2003 and 2006 caused a high shoot mortality and a steep decline in *P. oceanica* density (Marbà & Duarte, 2010). Among the remaining species, maximum projected summer temperatures by 2100 remain below albeit close to the upper LT_50_ limits and beyond the TPB of these species observed during our experiment. Moreover, seawater temperature may exceed the average SST projected for the end of this century during heat waves, which are expected to intensify and become more frequent (Frölicher, Fischer, & Gruber, 2018; Jordà et al., 2012). Then, the persistence/recovery of these populations will largely depend on the time window between consecutive heat wave events that compromise species survival. Finally, we found that *C. compressa* displayed relatively low thermal sensitivity on both the rising and falling phases. Moreover, *C. compressa* has shown to be resistant to the coupling of multiple anthropogenic stressors (including seawater warming and associated interspecific interactions) and show no signs of regression in areas where forests of other members of *Cystoseira* genus disappeared, such as the Italian coast, Adriatic Sea (Perkol‐Finkel & Airoldi, 2010), and in Albères coast, France (Thibaut et al., 2005). Therefore, despite current summer temperatures exceed its *T*_opt_, our findings suggest that *C. compressa* may be resistant to warming in the short term.

The comparisons between the upper thermal limits of experimental populations from Mallorca with the temperature range experienced by these six species across their global distribution revealed insights about population patterns in thermal physiology. If a viable population of a species occurs in a particular location, then it can be inferred that the thermal limits of that population exceed the upper temperatures experienced at that location. The upper thermal limits of both *H. tuna* and *P. oceanica* observed in the current study were lower than temperatures experienced by some of their conspecific populations. This could reflect seasonal acclimation (see above) or suggest that there may be population variation in thermal limits (e.g., genetic adaptation) within each of these species. Considering that *H. tuna* is a thermophilic species, it has been shown that the evolutionary niche dynamics of the Mediterranean population differ considerably from other populations and other species of the *Halimeda* genus, allowing to inhabit areas with temperatures as low as 10°C (Verbruggen et al., 2009). Phylogenetic studies demonstrate that populations of *H. tuna* from the Mediterranean Sea are “paleo‐endemic” and not recent invaders from the Atlantic Ocean (Verbruggen, De Clerck, Schils, Kooistra, & Coppejans, 2005), which allowed this evolutionary distinction between other conspecific populations to take place. Evolutionary niche dynamics have not been examined for *P. oceanica*, but studies on the genetic structure of *P. oceanica* suggest a clear distinction of populations between western and eastern Mediterranean Sea (Chefaoui, Duarte, & Serrão, 2017), which may influence the thermal niche of these populations. For instance, in the eastern Mediterranean Sea and particularly in Cyprus, summer SSTs reach as high as 30°C, which are well above the LT_50_ of *P. oceanica* in our study using western Mediterranean plants. In Cyprus, *P. oceanica* thrives well in depths ranging from 0 to 40 m throughout the perimeter of the island and represents the easternmost reported population of the species (Telesca et al., 2015) with the highest frequency of unique private alleles (Arnaud‐Haond et al., 2007; Chefaoui et al., 2017).

Experimental upper thermal limits of the remaining four species were greater than the upper temperatures experienced by conspecific populations across their respective global distributions, suggesting niche underfilling by these species (Sunday, Bates, & Dulvy, 2012). Therefore, without additional empirical evidence of the thermal limits of conspecific populations, it is not possible to make further inferences about intraspecific similarities or differences in thermal limits for these species. Nevertheless, it is noteworthy that there is evidence for genetic divergence between conspecific populations either at local or regional scales for *C. nodosa*,* C. prolifera,* and *P. pavonica* (Alberto et al., 2008; Tomasello et al., 2009; Silberfeld et al., 2013; Elena Varela‐Álvarez et al., 2015), making it plausible for the existence of different fundamental niches and the emergence of local ecotypes (Ackerly, 2003; Ehlers, Worm, & Reusch, 2008; Pakker & Breeman, 1994) with the capacity of tolerating different thermal limits.

In conclusion, our results demonstrate how the thermal niche of Mediterranean macrophytes differs among species within a single location and points to potentially variable responses to warming among species within coastal marine ecosystems. *C. nodosa* was the species that appeared likely to benefit from global warming over the coming decades. For the remaining five species, ongoing warming may have negative effects on their fitness over the coming decades. *P. oceanica* appeared as the most vulnerable to warming, which raises concerns given its critically important role for the structure, provision of ecosystem services (e.g., carbon sequestration, food web, coastal protection), and resilience of coastal Mediterranean ecosystems. Future studies looking at genotypic variation and or geographic patterns of conspecific populations in thermal tolerance will be useful to further understand how these local patterns relate to the global distribution and thermal sensitivity of these species.

## CONFLICT OF INTEREST

The authors have no conflict of interest to declare.

## AUTHORS CONTRIBUTION

IS, SB, GR, and NM conceived this study. IS and GR performed experimental work. IS analyzed experimental data. GJ acquired SST data. All authors wrote the manuscript.

## DATA ACCESSIBILITY

Data accessible at Digital CSIC URI: https://hdl.handle.net/10261/170999 (Savva et al., 2018)
