# Supplementary material for: Thermal tolerance of Mediterranean marine macrophytes: Vulnerability to global warming
Source: Ecol Evol. 2018 Nov 11;8(23):12032–43. doi: 10.1002/ece3.4663 (PMC6303755; doi:10.1002/ece3.4663)

Thermal tolerance of Mediterranean marine macrophytes: vulnerability to global warming.

Ioannis Savva^1, 3^, Scott Bennett^1^, Guillem Roca^1^, Gabriel Jordà^2^, Núria Marbà^1*^

^1^ Global Change Research Group, Institut Mediterrani d’Estudis Avançats (CSIC-UIB), Miquel Marquès 21, 07190 Esporles, Spain

^2^ Marine Ecosystem Dynamics Group, Institut Mediterrani d’Estudis Avançats (CSIC-UIB), Miquel Marquès 21, 07190 Esporles, Spain

^3^ Marine & Environmental Research (MER) Lab Ltd, Limassol, 4533, Cyprus

* corresponding author: telephone: +34 971611720; FAX: +34 971 611761; e-mail: [nmarba@imedea.uib-csic.es](mailto:nmarba@imedea.uib-csic.es)

## Maximum quantum yield

## Materials and Methods

The maximum quantum yield (MQY), a proxy of the photosynthetic efficiency, was determined as the *in vivo* chlorophyll a of the photosystem II (PII), using a pulse amplitude modulated (PAM) fluorometer (Waltz Diving-PAM).

Young parts of seagrass leaves and macroalgal laminae/thalli underwent 15 minutes of dark-acclimation (sufficient to allow plant relaxation and opening of all PSII) using specialized leaf clips. Once all the fragments were dark-acclimated, the maximum photosynthetic yield was determined (n=7) after initiating a quasi-darkness measurement (~1.5 μmol photons m^-2^ s^-1^) followed by a saturating light, to provide estimates of the ground fluorescence (Fo) and the maximum fluorescence (Fm) respectively, under fully reduced PII reaction centres (Maxwell and Johnson 2000). The MQY was calculated as follows:

$$Maximum quantum yield =\frac{Fm-Fo}{Fm}$$

where, *Fo* is the ground fluorescence in darkness and *Fm* the maximum fluorescence in darkness obtained prior and post saturating light, respectively.

# The relationship of MQY with temperature was statistically tested on the rise and falling phase of the performance curves through linear regression as part of the warming sensitivity analysis. To assess if temperature had an immediate effect on the photosynthetic efficiency, the responses of MQY across treatments at the beginning and at the end of the experiment were tested through a two-way analysis of variance with temperature and time being the contributing factors. Assumptions for normality and equal variances were verified using the Anderson-Darling and Bartlett tests, respectively. When assumptions of normality or homogeneity were not met, data were transformed (log_10_ or square root). The level of significance α was adjusted to 0.05 for all statistical analyses, and all statistical analyses were conducted in R (R core team). All graphics were generated with R-studio, package: ggplot2 (Wickham 2009).

# Results

The effect of temperature on MQY did not differ at the beginning and at the end of the experiment across each temperature treatment for *C. compressa* (2-way ANOVA, df= 139, F = 1, *p* > 0.05), *C. prolifera* (2-way ANOVA, df =139, F = 1.83, *p* > 0.05) and *H. tuna* (2-way ANOVA, df = 139, F = f = 1.04, *p* > 0.05), suggesting that temperature had an immediate effect on the photosynthetic performance regardless of time. On the other hand, the interaction of temperature with time played a critical role in altering the MQY across the temperatures tested for *C. nodosa* (2-way ANOVA, df= 139, F = 3.15, *p* < 0.05), *P. pavonica* (2-way ANOVA, df = 139, F = 1.97, *p* < 0.05) and *P. oceanica* (2-way ANOVA, df = 139, F = 3.24, *p* < 0.05).

The bell shaped responses and the T_opt_ were similar to those of RGR, while the TPB of photosynthetic efficiency was wider. The photosynthetic efficiency of *P. oceanica* was maximal at 23.6 °C and it was the species to exhibit the widest TPB (12-30 °C) with no temperature induced stress on the colder limits when compared to other species (Table S2; Figure S3). This was followed by *H.* tuna, *P. pavonica* and *C. compressa* with TPB at 18-29.4 °C, 16.2-27.2 °C and 19.2-30.9 °C (Table S2; Figure S3). The photosynthetic efficiency of the former peaked at 25 °C, while for the two latters at 21.6 °C (the lowest in respect to the other species) and at 25.5 °C, respectively (Table S3). *C. prolifera* and *C. nodosa* were the two macrophytes to have their TPB in warmer temperatures. The former had the narrowest TPB (24.9-31.3 °C) overall with T_opt_ at 29.9 °C and the latter was the species with none thermal induced stress on the warmest range tested, having maximal photosynthetic efficiency at 29.04 °C and TPB at 21.4-34 °C (Table S2; Figure S3).

Overall, the activation energies acquired in this study from the MQY were less temperature-dependent than the RGR (Table S2; Figure S4), except from *C. prolifera* and *H. tuna* at the falling phase.

Table S1. Experimental information on the date of field collection, duration of acclimation, date of the beginning and end of the experiment and the in situ temperature during the sampling for each species.

| **Species** | ***P. oceanica*** | ***C. nodosa*** | ***C. compressa*** | ***C. prolifera*** | ***H. tuna*** | ***P. pavonica*** |
| --- | --- | --- | --- | --- | --- | --- |
| Date of field collection | 01/02/2016 | 01/02/2016 | 15/03/2016 | 15/03/2016 | 14/04/2016 | 14/04/2016 |
| Acclimation period (days) | 7 | 7 | 7 | 7 | 7 | 7 |
| Date of experiment start | 08/02/2016 | 08/02/2016 | 23/03/2016 | 23/03/2016 | 22/04/2016 | 22/04/2016 |
| Date of experiment end | 04/03/2016 | 04/03/2016 | 05/04/2016 | 05/04/2016 | 08/05/2016 | 06/05/2016 |
| Experiment duration (days) | 25 | 25 | 13 | 13 | 16 | 14 |
| *In situ* temperature (°C) | 18 | 18 | 17 | 16 | 17 | 17 |
| Ambient experimental chamber temperature (°C) | 10 | 10 | 10 | 10 | 10 | 10 |

**Table S2.** The temperature associated parameters (T_opt_, T_min_, T_max_, MQY_opt_) ± SE obtained by the Temperature Cardinal Model with Inflexion (CTMI), thermal performance breadth (TPB), the rate of change per degree of Celsius ± SE (Rate of rise, Rate of fall) and activation energy (Ea_rise_, Ea_fall_) ± SE of the rising and falling phase of the bell-shaped MQY responses for all 6 species. Note: asterisks indicate the significance of the CTMI parameters’ estimates (** p < 0.01, * p < 0.05); ns = not significant; N/A = not applicable, indicating unrealistic estimated values due to the lack of further empirical data below 12 °C and beyond 34 °C.

| **Temperature parameters** | ***C. nodosa*** | ***P. oceanica*** | ***C. prolifera*** | ***C. compressa*** | ***H. tuna*** | ***P. pavonica*** |
| --- | --- | --- | --- | --- | --- | --- |
| T_opt_ (°C) | 29.04 ± 2.5** | 23.6 ± 2.04** | 29.9 ± 0.8** | 25.5 ± 1.3** | 25 ± 0.9** | 21.6 ± 0.9** |
| T_min_ (°C) | N/A | N/A | N/A | N/A | N/A | N/A |
| T_max_ (°C) | N/A | 35.0 ± 0.7** | 34.6 ± 0.4** | N/A | 34.1 ± 0.4** | N/A |
| MQY_opt_ | 0.8 ± 0.03** | 0.7 ± 0.02** | 0.5 ± 0.02** | 0.6 ± 0.02** | 0.3 ± 0.02** | 0.5 ± 0.02** |
| TPB (°C) | 21.4-34 | 12-30 | 24.9-31.3 | 19.2-30.9 | 18-29.4 | 16.2-27.2 |
| Rate of rise (°C^-1^) | 0.0204 ± 0.00500 (R^2^ = 0.26; *p* < 0.05; N = 49) | 0.0011 ± 0.00240 (ns) | 0.0220 ± 0.00220 (R^2^=0.65; *p* < 0.05; N=56) | 0.0094 ± 0.00330 (R^2^=0.17; *p* < 0.05; N=43) | 0.0115 ± 0.00240 (R^2^=0.36; *p* < 0.05; N=43) | 0.0150 ± 0.00430 (R^2^=0.33; *p* < 0.05; N=29) |
| Rate of fall (°C^-1^) | 0.0260 ± 0.01200 (ns) | -0.0113 ± 0.0090 (ns) | -0.0700 ± 0.02000 (R^2^=0.37; *p* < 0.05; N=21) | -0.0019 ± 0.00610 (ns) | -0.0450 ± 0.00600 (R^2^=0.65; *p* < 0.05; N=36) | -0.0135 ± 0.02900 (R^2^=0.30; *p* < 0.05; N=50) |
| Ea_rise_ (eV) | 0.214 ± 0.410 (R^2^= 0.33; *p* < 0.05; N = 54) | 0.046 ± 0.036 (ns) | 0.600 ± 0.080 (R^2^= 0.50; *p* < 0.05; N = 54) | 0.157 ± 0.053 (R^2^= 0.18; *p* < 0.05; N = 43) | 0.337 ± 0.072 (R^2^= 0.35; *p* < 0.05; N = 43) | 0.246 ± 0.068 (R^2^= 0.33; *p* < 0.05; N = 29) |
| Ea_fall_ (eV) | 0.260 ± 0.14 (R^2^ = 0.17; *p* > 0.05; N = 21) | 0.452 ± 0.137 (R^2^=0.21; *p* < 0.05; N=42) | 2.084 ± 0.611 (R^2^=0.38; *p* < 0.05; N=21) | 0.049 ± 0.097 (ns) | 2.080 ± 0.331 (R^2^=0.52; *p* < 0.05; N=31) | 0.258 ± 0.062 (R^2^=0.26; *p* < 0.05; N=50) |

Table S3. The initial and final dry weights (DW g) with standard error (SE) of all species from across the temperature treatments.

| ***P. oceanica*** | | | | |  | ***C. nodosa*** | | | | |
| --- | --- | --- | --- | --- | --- | --- | --- | --- | --- | --- |
| Temperature (°C) | Initial DW (g) | SE | Final DW (g) | SE |  | Temperature (°C) | Initial DW (g) | SE | Final DW (g) | SE |
| 12 | 1.67 | 0.26 | 1.78 | 0.28 |  | 12 | 0.09 | 0.01 | 0.09 | 0.01 |
| 15 | 2.41 | 0.15 | 2.57 | 0.17 |  | 15 | 0.10 | 0.01 | 0.11 | 0.01 |
| 18 | 2.29 | 0.42 | 2.42 | 0.40 |  | 18 | 0.10 | 0.01 | 0.10 | 0.01 |
| 21 | 2.29 | 0.35 | 2.50 | 0.37 |  | 21 | 0.13 | 0.02 | 0.14 | 0.01 |
| 24 | 2.10 | 0.29 | 2.37 | 0.30 |  | 24 | 0.10 | 0.01 | 0.11 | 0.01 |
| 26 | 2.34 | 0.46 | 2.76 | 0.47 |  | 26 | 0.14 | 0.01 | 0.17 | 0.02 |
| 28 | 2.67 | 0.41 | 3.09 | 0.46 |  | 28 | 0.09 | 0.01 | 0.12 | 0.01 |
| 30 | 1.89 | 0.16 | 2.08 | 0.18 |  | 30 | 0.11 | 0.01 | 0.14 | 0.01 |
| 32 | 1.75 | 0.14 | 1.83 | 0.15 |  | 32 | 0.10 | 0.01 | 0.12 | 0.01 |
| 34 | 2.18 | 0.22 | 2.21 | 0.25 |  | 34 | 0.11 | 0.01 | 0.13 | 0.01 |
|  |  |  |  |  |  |  |  |  |  |  |
| ***C. compressa*** | | | | |  | ***C. prolifera*** | | | | |
| Temperature (°C) | Initial DW (g) | SE | Final DW (g) | SE |  | Temperature (°C) | Initial DW (g) | SE | Final DW (g) | SE |
| 12 | 0.18 | 0.02 | 0.23 | 0.03 |  | 12 | 0.09 | 0.01 | 0.08 | 0.01 |
| 15 | 0.19 | 0.02 | 0.26 | 0.03 |  | 15 | 0.08 | 0.01 | 0.07 | 0.01 |
| 18 | 0.18 | 0.02 | 0.26 | 0.03 |  | 18 | 0.10 | 0.02 | 0.10 | 0.02 |
| 21 | 0.20 | 0.03 | 0.29 | 0.04 |  | 21 | 0.08 | 0.01 | 0.09 | 0.01 |
| 24 | 0.19 | 0.03 | 0.32 | 0.06 |  | 24 | 0.08 | 0.01 | 0.11 | 0.01 |
| 26 | 0.21 | 0.03 | 0.33 | 0.04 |  | 26 | 0.10 | 0.02 | 0.13 | 0.02 |
| 28 | 0.16 | 0.01 | 0.26 | 0.02 |  | 28 | 0.10 | 0.02 | 0.13 | 0.01 |
| 30 | 0.17 | 0.02 | 0.26 | 0.02 |  | 30 | 0.07 | 0.00 | 0.09 | 0.00 |
| 32 | 0.16 | 0.03 | 0.24 | 0.04 |  | 32 | 0.09 | 0.01 | 0.09 | 0.01 |
| 34 | 0.20 | 0.04 | 0.26 | 0.05 |  | 34 | 0.08 | 0.01 | 0.08 | 0.01 |
|  |  |  |  |  |  |  |  |  |  |  |
| ***P. pavonica*** | | | | |  | ***H. tuna*** | | | | |
| Temperature (°C) | Initial DW (g) | SE | Final DW (g) | SE |  | Temperature (°C) | Initial DW (g) | SE | Final DW (g) | SE |
| 12 | 0.09 | 0.02 | 0.10 | 0.03 |  | 12 | 0.02 | 0.00 | 0.02 | 0.00 |
| 15 | 0.09 | 0.03 | 0.11 | 0.03 |  | 15 | 0.02 | 0.00 | 0.03 | 0.00 |
| 18 | 0.10 | 0.02 | 0.13 | 0.03 |  | 18 | 0.02 | 0.00 | 0.03 | 0.00 |
| 21 | 0.10 | 0.02 | 0.13 | 0.03 |  | 21 | 0.02 | 0.00 | 0.03 | 0.00 |
| 24 | 0.10 | 0.02 | 0.14 | 0.03 |  | 24 | 0.03 | 0.00 | 0.03 | 0.00 |
| 26 | 0.12 | 0.03 | 0.15 | 0.03 |  | 26 | 0.03 | 0.01 | 0.03 | 0.01 |
| 28 | 0.12 | 0.02 | 0.16 | 0.03 |  | 28 | 0.03 | 0.00 | 0.03 | 0.01 |
| 30 | 0.15 | 0.03 | 0.18 | 0.03 |  | 30 | 0.03 | 0.00 | 0.03 | 0.00 |
| 32 | 0.11 | 0.01 | 0.12 | 0.01 |  | 32 | 0.02 | 0.00 | 0.02 | 0.00 |
| 34 | 0.11 | 0.02 | 0.11 | 0.02 |  | 34 | 0.02 | 0.00 | 0.02 | 0.00 |

Table S4. The upper SST of the species’ global distribution, experimental LT_50_ and the thermal buffer for each species. Thermal buffer calculations *for P. oceanica, C. prolifera, H. tuna* and *P. pavonica* were calculated as the difference between their LT_max50_ and local T_max_ (27.6 °C), whereas for *C. nodosa* and *C. compressa*, as the difference between the maximum experimental temperature (34 °C) and local T_max_.

|  | ***P. oceanica*** | ***C. nodosa*** | ***C. compressa*** | ***C. prolifera*** | ***H. tuna*** | | ***P. pavonica*** | |  |
| --- | --- | --- | --- | --- | --- | --- | --- | --- | --- |
| Upper SST of global distribution (°C) | 30.0 | 30.2 | 30.0 | 31.3 | | 32.2 | | 34.7 | |
| Experimental LT_50_  (°C) | 28.9 | > 34 | > 34 | 33.4 | | 31.8 | | 35.3 | |
| Thermal buffer (°C) | 1.3 | 6.4 | 6.4 | 5.8 | | 4.2 | | 7.7 | |


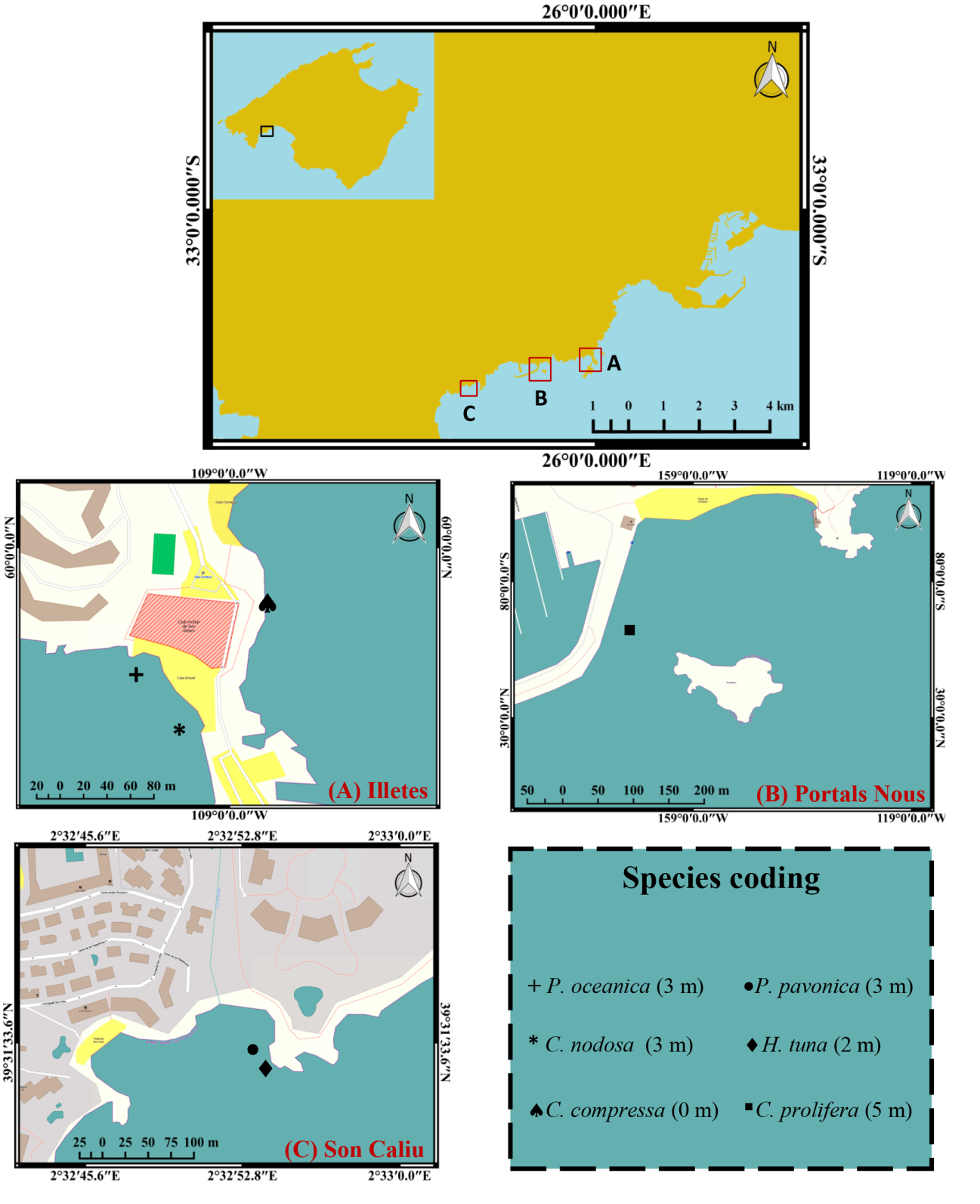


Figure S1. The precise sampling sites (A, B, and C) located in the south-west Mallorca island, demonstrating where each species has been collected and at which depth. Note: *P. oceanica* and *C.nodosa* were collected from a sandy floor, *C. compressa* from a rocky shore, *C. prolifera* from a *P. oceanica* mat and rocky crevice, *P. pavonica* and *H. tuna* from a rocky reef.


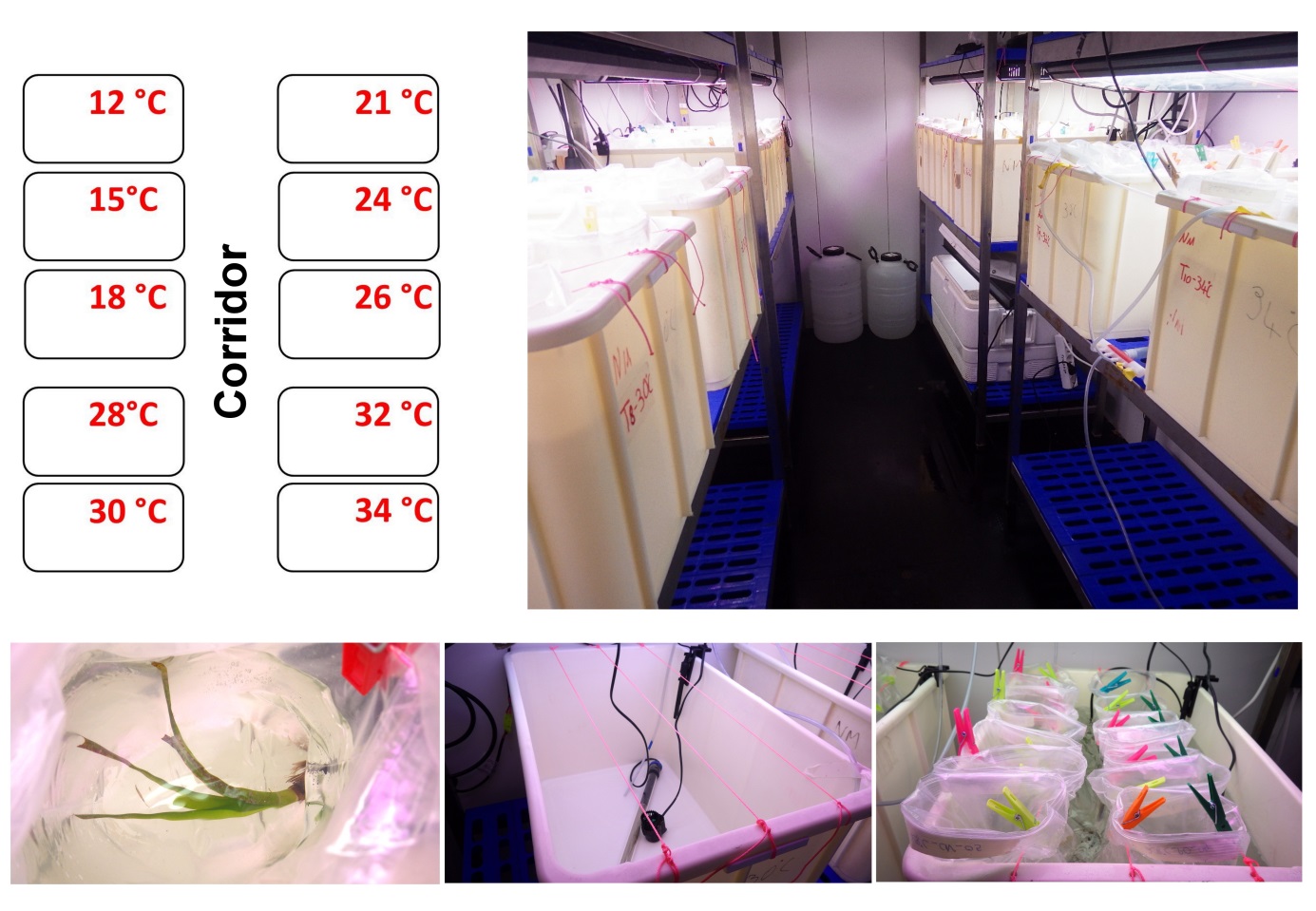


Figure S2. The experimental scheme (A) and setup (B), illustrating the 10 baths at different temperatures (B), a bath incubating 14 double layered plastic bags (E), the content of a double layered plastic bag (C) and the associated heating, stirring and temperature recording systems (D).

**A. Experimental scheme**

**B**

**C**

**D**

**E**

Figure S3. The bell-shaped MQY responses of all six macrophyte species to experimental seawater warming fitted with the CTMI. The shaded area indicates the upper and lower 95% confidence intervals. Vertical dotted lines illustrate the TPB.


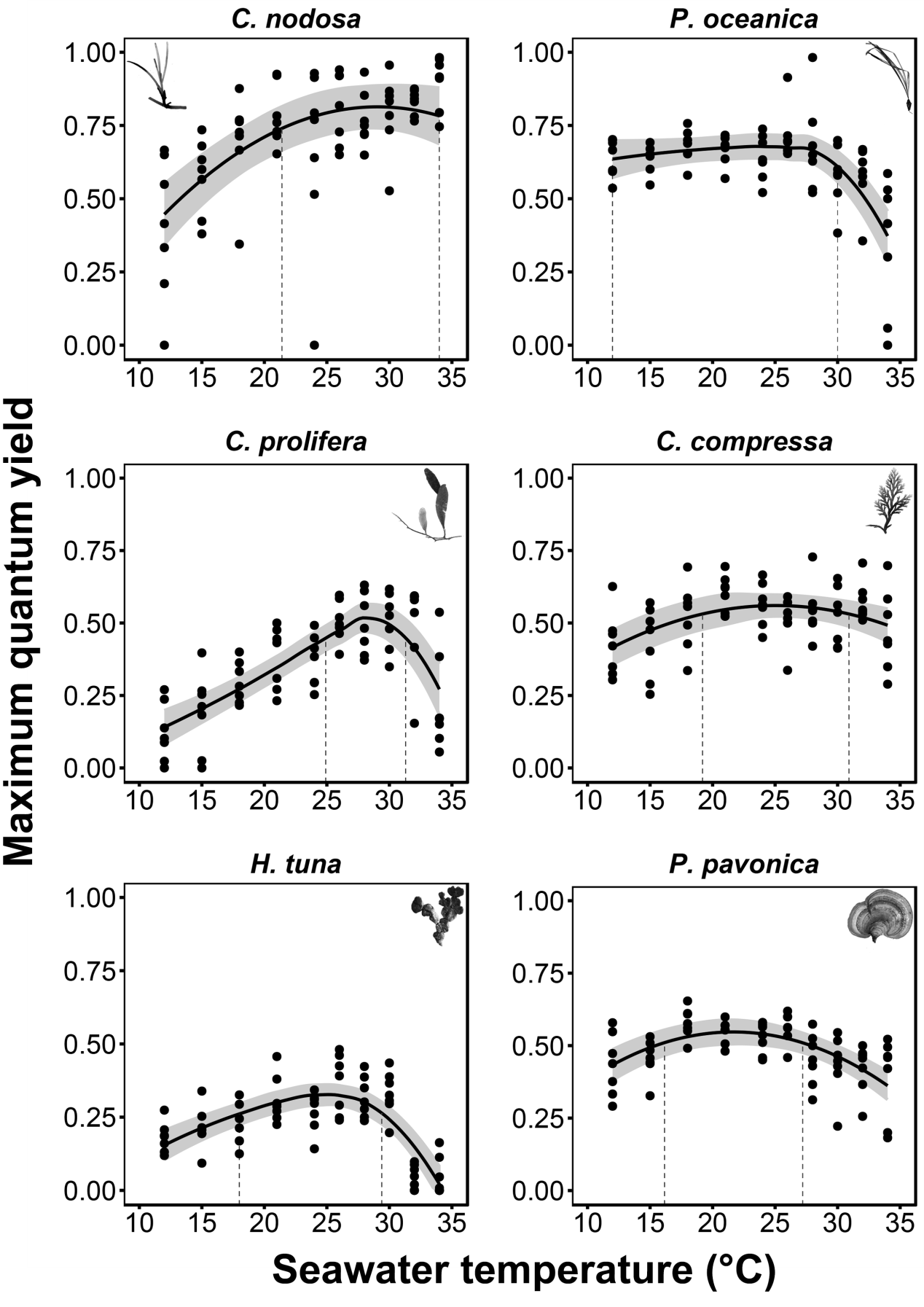


Figure S4. The activation energy at the falling phase (Ea_fall_) against the rising phase (Ea_rise_) of all six macrophytes for MQY. The black diagonal line represents a 1:1 ratio line. PO: *P. oceanica*, CN: *C. nodosa*, CYS: *C. compressa*, CP: *C. prolifera*, HT: *H. tuna* and PP: *P. pavonica*.


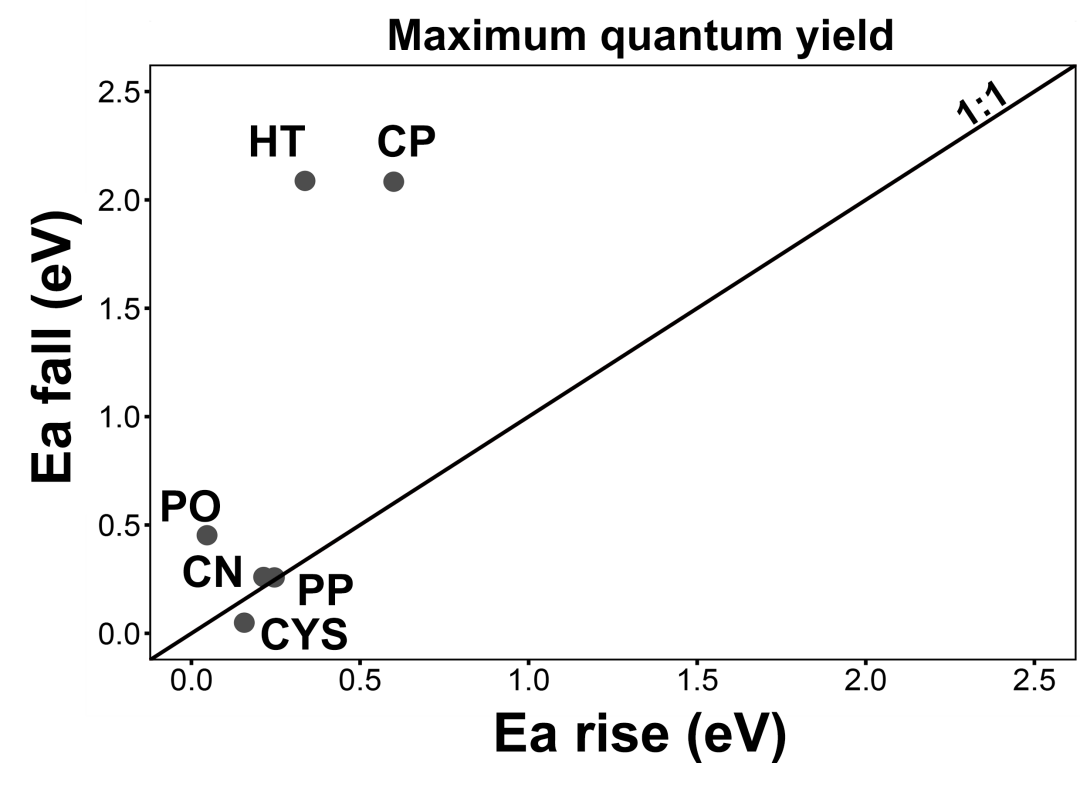


Figure S5. Specimens with healthy condition (left column) and specimens with tissue necrosis/meristem mortality (right column) of five out of the six species. Scale equals to 1 cm.


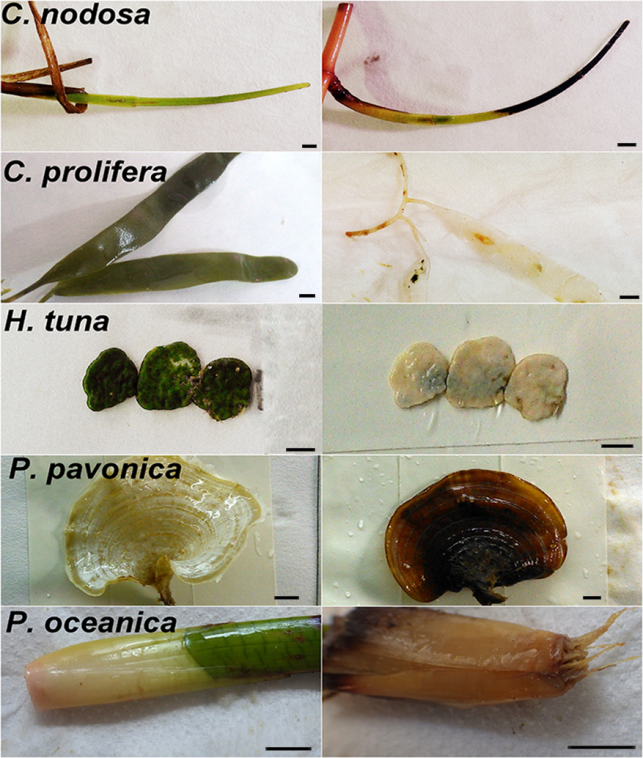

Supplement: Supplementary file 1 [file ECE3-8-12032-s001.docx]
